# A rapid qualitative assessment of barriers associated with demand and uptake of health facility-based childhood immunizations and recommendations to improve immunization service delivery in Sokoto State, Northwest Nigeria, 2017

**DOI:** 10.11604/pamj.supp.2021.40.1.23793

**Published:** 2021-11-16

**Authors:** Neetu Abad, Belinda Vernyuy Uba, Palak Patel, David Nyampa Barau, Osigwe Ugochukwu, Nuruddeen Aliyu, Halimatu Bolatito Ayanleke, Richard Franka, Ndadilnasiya Endie Waziri, Omotayo Bolu

**Affiliations:** 1 Global Immunization Division, US Centers for Disease Control and Prevention, Atlanta, Georgia, United States of America,; 2 Nigeria STOP Polio Program, Abuja, Nigeria,; 3 ORISE (Oak Ridge Institute for Science and Education) Fellow, Global Immunization Division, US Centers for Disease Control and Prevention, Atlanta, Georgia, United States of America,; 4 State Emergency Routine Immunization Coordinating Centre, Sokoto, Nigeria

**Keywords:** Nigeria, immunization, vaccine demand, vaccine hesitancy

## Abstract

**Introduction:**

This rapid qualitative assessment aimed to understand factors associated with persistent low vaccination demand and uptake, and recommendations to improve health facility-based childhood immunization services in Sokoto State, Nigeria.

**Methods:**

In 2017, 20 focus group discussions and 16 in-depth interviews were conducted with administrative personnel, healthcare workers, caregivers, and community influencers across three local government areas in Sokoto state, Northwest Nigeria. Participants were purposefully selected to capture a range of perspectives regarding access to health services, campaign- and facility-based immunizations, confidence in immunizations, and recommendations to improve childhood immunization uptake.

**Results:**

One hundred and ninety-three individuals participated in the assessment. Commonly reported barriers to receiving childhood immunizations include: inadequacy of health services to meet community needs, preference for campaign vs. facility-based immunizations, the negative influence of rumors and misinformation, and opposition to vaccines among male heads of households. Recommendations to improve uptake of childhood immunizations include: improving immunization service delivery in health facilities, involving community leaders in building demand for immunization, and providing access to free health services and non-cash incentives.

**Conclusion:**

Rapid assessment results highlight community, facility, and administrative barriers associated with low demand for and uptake of health facility-based childhood immunizations and offer recommendations to improve immunization services in Sokoto state, Nigeria. Findings demonstrate the persistence of service and supply side barriers such as infrastructure and personnel issues, but also highlight the influence of behavioral factors such as low prioritization of receiving childhood immunizations, misinformation, and gender dynamics on whether communities accept or seek out immunization services.

## Introduction

Optimal childhood immunization coverage resulting in control, elimination and eradication of vaccine-preventable diseases is a major goal of the Expanded Program on Immunization (EPI) [1]. The Global Vaccine Action Plan 2011-2020 (GVAP) recognizes that inadequate vaccine supply, access barriers, and low demand are common challenges facing immunization programs at the national, district, and sub-district levels [2]. Gaps in immunization coverage persist between high-income and low-and-middle income countries. For instance, coverage of the third dose of diphtheria-tetanus-pertussis containing vaccine (DTP3) for infants under a year old is lowest in Africa (74%) compared to other regions of the world (> 80%) [2]. National DTP3 coverage is often used as a proxy for vaccination coverage and performance [3]. Nigeria, in particular, has struggled to achieve the GVAP goals of ≥ 90% national coverage and ≥ 80% coverage in every district for all vaccines in a country´s national immunization schedule by 2020 [2]. Nigeria continued to report circulation of vaccine-derived poliovirus, and has one of the highest under-five mortality rates in the world (109 child deaths per 1,000 live births in 2015); approximately one in four of those deaths are preventable if children received all recommended childhood immunizations [4]. Additionally, DTP3 coverage in Nigeria decreased from 38% in 2013 to 33% in 2016 [5].

Sokoto state in northwest Nigeria has consistently reported very low DTP3 coverage. In 2016, DTP3 coverage in Sokoto state was 3%, as compared to 14% median state coverage elsewhere in the country [5]. In recent years, there have been intensified efforts by government and partners to improve supply side constraints of vaccination coverage in Nigeria [6,7]. However, vaccination coverage in many parts of the country, particularly in northwest Nigeria, has not met the desired targets [8]. Past published reports exploring factors for low demand for immunizations such as polio and measles in Nigeria highlight several potential barriers, including rumors about vaccines, fears of adverse events following immunizations, and lack of trust in vaccinations and health staff [9-14]. Similar factors are thought to also be associated with low demand for all childhood immunizations, particularly those offered primarily in health facilities, but this issue has not been explored in detail. To better understand the contribution of vaccine demand and acceptance to the persistent gaps in immunization coverage among children in Sokoto state, the U.S. Centers for Disease Control and Prevention (CDC) partnered with the Nigeria STOP Polio Program (NSTOP) to conduct a rapid qualitative assessment of barriers associated with uptake of health facility-based childhood immunization and recommendations to improve delivery of immunization services.

## Methods

### Design

The rapid qualitative assessment included both focus group discussions (FGDs) and in-depth interviews (IDIs) across three purposefully selected local government areas (LGAs) in Sokoto state, northwest Nigeria: Shagari, Dange Shuni, Illeila. Shagari and Illeila are characterized as rural, while Dange Shuni is characterized as urban. These LGAs were selected if they reported DTP3 coverage below 20%, were geographically diverse, and were suitable for future program intervention [15]. The rapid assessment was funded by CDC through a cooperative agreement with the Africa Field Epidemiology Network (AFENET). FGD participants included female caregivers and male heads of households, community ward development committee members, health facility-based immunization service providers (locally referred to as routine immunization service providers), and LGA administrative staff in each LGA. IDI participants included the district head, male and female community influencers as identified by the community, including traditional birth attendants, farmers, housewives, and traders. These categories of participants were selected to capture a wide range of factors related to vaccine demand and acceptance, as well as recommendations for improving childhood immunization services in Sokoto state. All participants provided information about their age, gender, occupation, education, and religion.

### Instruments

CDC and NSTOP developed FGD and IDI topic guides to ask participants about their access to health services, experience with campaign-based and health facility-based vaccinations, perceptions of barriers to uptake of childhood immunizations, trust and confidence in immunizations, and recommendations to improve uptake of childhood immunizations in their community. These guides were reviewed by the field team, pre-tested, and translated on paper from English into Hausa, the primary language of the selected LGAs in Sokoto state.

### Training and data collection

Twelve residents and three field supervisors from the Nigeria Field Epidemiology and Laboratory Training Program were recruited and trained in qualitative data collection, transcription, and analysis. In June 2017, the field team deployed to Sokoto to conduct qualitative interviews. Each of the three LGA rapid assessment teams were comprised of one field supervisor and two interview teams. Each team contained one moderator and one note-taker. In accordance with cultural norms in Sokoto, female and male caregivers were interviewed by all female and all male interview teams, respectively. Administrators and healthcare workers were interviewed by mixed gender groups. Each FGD or IDI was conducted by a designated moderator, and the note-taker took notes in accordance with a predesigned interview template corresponding to interview topic guides. Each LGA team completed 3-4 FGDs or IDIs per day. At the end of each day, the field supervisor held debriefing meetings with their LGA teams where they reviewed notes from each interview conducted that day to identify emerging themes and key observations and discussed ways to improve subsequent data collection. Following the debriefing meetings, supervisors synthesized emerging barriers to receiving childhood immunizations and recommendations to improve uptake. All interviews were audio recorded and transcribed by data collectors in the local language, Hausa, and then translated to English by data collectors.

### Data analysis

A senior behavioral scientist supervised the data collection process and drafted an initial codebook based on topics included in the interview guides. An independent research assistant coded an initial set of interview transcripts, utilizing grounded theory [16] to revise the primary codebook and identify sub-codes. A sample of transcripts were reviewed and coded by both researchers, any coding discrepancies were discussed and resolved, and the codebook was updated. The remaining transcripts were coded based on the updated codebook. Coding and analysis were conducted using Dedoose Version 7.0.23, a web-based qualitative and mixed method research data software. The coded themes reflect primary barriers to receiving childhood immunizations, as well as recommended strategies to improve uptake. The analysis was inductive in that the team identified themes based on questions and probes in the interview guides. Additional themes were deductively identified based on emerging topics from the interviews not included in the guides.

## Results

The Sokoto state rapid assessment conducted 20 FGDs and 16 IDIs, which included 193 participants. Table 1 lists the categories of rapid assessment participants by the type and number of interviews conducted as well as overall number of participants. The age range was 18-80 years; median 35 years. All participants reported Islam as their primary religion, and majority of caregivers reported Islamic education as their primary source of education. The most commonly reported occupations among caregivers were farming, trading and cattle-rearing.

**Table 1:** qualitative interviews by participant type

Type of Participant	Type of Interview	Number of Interviews	Number of Participants	Median Age in years (Range)	Gender
District Head	IDI	3	3	60 (59-65)	Male
Ward Development Committee Focal Person	IDI	3	3	31 (29-50)	Male
Routine Immunization Focal Person on Ward Development Committee	IDI	2	2	33 (32-34)	Mixed
Male Community Influencers	IDI	4	4	60 (45-80)	Male
Female Community Influencers	IDI	4	4	50 (34-55)	Female
Local Government Administration team	FGD	3	21	39 (29-65)	Mixed
Routine Immunization Service Providers	FGD	3	25	32 (24-40)	Mixed
Ward Development Committee	FGD	3	26	49 (30-80)	Male
Mothers	FGD	6	57	25 (18-60)	Female
Heads of Households	FGD	5	48	40 (23-78)	Male
Total		36 (20 FGDs; 16 IDIs)	193	35 (18-80)	

### Barriers associated to receiving health facility-based childhood immunizations

Primary barriers to receiving childhood immunizations as reported by rapid assessment participants are summarized below.

### Inadequate health services

The most frequently cited concern among all respondents was perceived inadequacy of health services, including lack of staff and poor interpersonal communication between healthcare workers and caregivers, and a lack of healthcare workers who were from local communities.

*If a person and his/her child goes to the hospital and (has) been treated nicely, it encourages one to go back. Not that I want to speak ill of health service providers but honestly the way they shout and scold people maybe because they didn´t bathe before going and are having odor. This are among the things that discourages them so health service providers should try and have regards for their patient...- District Head, Dange Shuni*

Male heads of households noted a desire for having more female immunization service providers, and objected to their wives interacting with male immunization service providers.

*You may find a health facility conducting ANC (antenatal care) services which are supposed to be carried out maybe by a female health worker, but due to some certain problems, a male health worker carries out such services; this makes it very difficult for the man to take his wife to a male for this kind of services. -LGA Team Dange Shuni*

*...If a male service provider comes and stays...round to give to them, we will not allow (women) to rush and go there. -Heads of households, Dange Shuni*

Other supply-side barriers cited by respondents included poor quality of health facility infrastructure, and challenges accessing health services due to factors such as distance, poor road conditions, limited transportation options, and lack of supplies at health facilities.

*The roads going to the health facility are not good, in one way or another. You may find some settlements with at least a distance of 20 - 30 kilometers before getting to the health facility. This is a challenge. - LGA team, Dange Shuni*

Routine immunization service providers acknowledged the reality of these challenges, but also noted that lack of salary and logistical complications such as the rainy season often impeded their work in communities.

*The final advice I will like to give is that there are service providers who have salary issues which have not been addressed. It will be good for the government to look into it. -Routine Immunization Service Providers, Dange Shuni*

*For instance, if one plans to go for an outreach during raining season, you may not meet people because they have gone to the farm...once the rain stopped, people went out. Now if you schedule the work at that time it will not be possible and you may have problem. -Routine Immunization Service Providers, Dange Shuni*

They also reported a lack of training in critical issues like improving their communication with caregivers.

*I: What training did you receive on communicating with caregivers?*

*R: None. RI Providers, Illeila*

### Perceptions of health facility-based immunization service delivery

All respondents reported widespread lack of community awareness of the benefits and availability of immunization services available at health facilities.

*For some people, they believe that the one given at home and the one given at the hospital is the same...after they have been given at home, they decide not to go to the hospital. - Male Heads of Households, Shagari*

Caregivers expressed fear of adverse events following immunization, particularly fever and pain. Some healthcare workers noted that caregivers felt vaccine-preventable diseases received more attention than other diseases that affected children, and caregivers reported that traditional medicine may be more accessible and effective in treating ailments, including vaccine-preventable diseases.

*But they saw that more attention is placed on these ones to the point that it is done even at homes for them while if their child is sick and taken to the hospital because of malaria or anything, one will have to buy the drugs with his money. -Routine Immunization Service Providers, Dange Shuni*

*I have received up to six of the tetanus injections but still there was no improvement, until when my relation down here bought herbs worth one thousand naira and then paid five hundred naira for weigh bill and after taking it, I got healed. -Heads of households, Dange Shuni*

### Vaccination rumors and refusals

The community reported various underlying reasons for actively refusing health facility-based immunization services including political affiliations, religious reasons, and objections by influential community or household members, particularly male spouses.

*Some people relate it to religion. They believe the Islamic religion forbids you from seeking medication before an ailment. -Heads of Households, Dange Shuni*

Several mothers reported that their husbands did not give permission for them to take their child to a health facility for vaccination.

*R: Honestly for me already my husband forbids it*.

*I: You have never asked your husband his reasons for refusing?*

*R: Honestly I have not asked what their reasons are. You know we don´t have any right over the child. -Mothers, Dange Shuni*

Service providers and community participants indicated that rumors about immunization were actively shared in communities and had an effect on perceptions towards immunization services. These rumors included beliefs that vaccines cause infertility, were meant to reduce the population size of their predominantly Islamic communities, and caused children to become truant and disobedient. Caregivers also reported that community members became suspicious of free vaccines given when other medical treatments cost money.

*Someone will say, why is he followed to his house with vaccine, whereas when he goes to the hospital, he is not given free drugs. He becomes skeptical about it. -Heads of Households, Shagari*

### Vaccination in health facilities vs. campaigns

Some service providers and community members discussed the influence of repeat door-to-door vaccination campaigns on community perceptions towards health facility-based immunization services. Participants generally believed that community members could not distinguish between vaccines offered at a health facility and those brought to their door.

*R: People take (vaccines delivered by campaign) with much importance than the routine immunization. For the former they will bring it to your house while the latter you have to go and meet them*.

*I: Do you think this stops them from going for routine immunization?*

*R: Yes...because for house to house, they don´t tell them to come out and go. Some feel the routine immunization and the house to house is the same so no need for them to go. -Community Influencer, Dange Shuni*.

Some caregivers reported that they associated outbreaks with the need to receive immunizations, which diminished recognition that immunizations are also needed to prevent vaccine-preventable diseases during non-outbreak periods.

*(It is) assumed that immunization is received when there is outbreak of disease and so the people get immunized against the disease to prevent infection. -Heads of households, Dange Shuni*

### Recommendations to increase uptake of childhood immunizations

Participants were asked to give recommendations on how uptake of childhood immunizations could be increased in their community. These recommendations can be grouped into three categories: improving the quality of immunization service delivery, strengthening social mobilization efforts, and provision of incentives to encourage uptake of childhood immunizations.

### Improve quality of immunization services offered at health facilities

Participants asked for greater investments in training, monitoring, and supervision of immunization service providers, particularly in the area of interpersonal communication skills. They also asked for improvements to the infrastructure of immunization services in facilities and offering of free or facilitated transportation of caregivers and their children.

*Firstly, the health worker must develop (interpersonal communication) skills so as to have a good human relationship with the people...so health workers need to develop human relationships. Secondly, session sites must be arranged during the session. For instance, if a fixed post is be carried out at the health facility, before the RI providers or caregivers arrive, a conducive sitting environment needs to be set in place for the comfort of the people, not that parents with children will come around and stand and a different person might be called in to be immunized while he/she is still standing. The service provider should have a knowledge of the antigen to be given at routine immunization. For instance, if he/she is to give BCG, DTP or OPV, he/she should know the site to be given. Being knowledgeable at his/her word, will help in getting a good turn-out at the health facility*.

*LGA Team, Dange Shuni*

### Strengthen social mobilization

Many participants recommended the need for widespread improvements to social mobilization to build awareness and appreciation for immunization services, and to counter vaccine refusals. Recommendations included mobilization of key influencers within communities such as traditional and religious leaders, traditional birth attendants, male barbers, district heads, and volunteer community mobilizers.

*The community leader, the religious leader, if the two speak with people and enlighten them about the importance and assure them of the harmless effect, then maybe they would accept it. - Head of Households, Dange Shuni*

Participants also encouraged facilitator-led health talks to be held at various places within the community and in health facilities.

### Provision of incentives

Many participants shared their belief that cash and non-cash incentives could motivate community members to take their children to health facilities for immunization services, particularly incentives that could increase quality of life. Suggested non-cash incentives for households included soap, sugar, Indomie noodles, sweets and clothes for children, milk, utensils, and boreholes. Several caregivers also asked for free paracetamol to help relieve fevers post-vaccination. Some participants expressed that while incentives might increase uptake of childhood immunizations, if stopped, uptake may drop to previously low levels or worse.

*There should be a provision for every child from zero to one year to be given free drugs in the hospital, apart from the immunization vaccine. With that every father will encourage the mother to be going for the exercise. That will also cover all forms of sickness that might come up during the immunization period, like fever, headache and so on. -Head of Households, Illeila*

## Discussion

This rapid qualitative assessment highlights factors associated with low demand for and uptake of health facility-based childhood immunizations, and generates recommendations for improving uptake of immunizations in three LGAs within Sokoto State, Northern Nigeria. Results demonstrate that administrative, healthcare worker, and community participants report many similar commonly observed barriers to receiving childhood immunizations, particularly the inadequacy of health services to meet community needs, preference for immunizations delivered via campaigns, fear of adverse events following immunization, and the influence of rumors on immunization uptake. Previous research has indicated longstanding service delivery barriers in northern Nigeria including poor supply chains, cold chain equipment, and physical infrastructure; inadequate number of health facilities; lack of capacity and accountability at local government levels; and human resource gaps [17-19]. Our findings show the persistence of some of these barriers but also highlight the influence of behavioral factors such as low prioritization of childhood immunization, misinformation, and gender dynamics on whether communities accept or seek out immunization services at health facilities.

### Low prioritization of childhood immunization

Many participants indicated that poor interpersonal communication between healthcare workers and caregivers, persistent training gaps among healthcare workers, and few available local healthcare workers facilitated the perception that health services do not meet community needs. Lack of basic community awareness of childhood immunization and its benefits demonstrates a communication gap between immunization services and local communities, and caregivers often had difficulty distinguishing between immunizations offered at health facilities and immunizations offered via campaigns during outbreaks. Disease-specific elimination strategies, such as the polio eradication initiative, have sought to vaccinate residents where they are in the community, either in their compounds or at outreach posts through campaigns [20,21]. This strategy has yielded success in reaching single antigen disease-specific goals, but it has not reinforced health seeking behavior at health facilities for immunization services or helped to reach fully immunized child health goals. The consequence of this may be evidenced in the low rates of childhood immunization uptake at fixed sites [13].

### Rumors

We documented rumors circulating in communities that contributed to caregivers actively or passively not seeking immunization services. Many of these rumors were also prevalent during polio eradication initiative strategies throughout northern Nigeria [14,21,22]. The content of these rumors is often similar, with some community members expressing belief that routine immunizations are covert family planning tools that cause infertility or sterility, or that vaccines are ‘suspicious’ because they are free. However, some new rumors emerged, such as the rumor that vaccines cause children to become disobedient. Also, similar to experiences during polio eradication efforts in Nigeria [22,23], community members expressed tension between their Islamic religious faith and willingness to receive immunizations, in that God is responsible for health and well-being, so there is no need to vaccinate to prevent disease.

### Gender dynamics

Our data indicate that many male heads of households opposed allowing their wives to seek immunizations for their children, or opposed to immunization in general. Some cited religious beliefs as incompatible with immunization, in that only God could prevent the onset of disease. Others cited health systems factors such as distrust of non-local healthcare workers or the lack of female healthcare workers in local health facilities to provide services to their wives and children. Female caregivers discuss their lack of autonomy regarding health care decision-making within the family as a barrier to getting their children immunized. Addressing sociocultural and health systems factors could create opportunities to address gender barriers to accessing immunization services in this part of Nigeria.

### Strategies to improve immunization service delivery

Community-generated recommendations to improve uptake of childhood immunizations call for improving health facility infrastructure and building community awareness and demand for childhood immunization. Enhancing social mobilization practices and including community and religious leaders also serve to bridge the divide between facilities and communities, and build on strategies that have been successfully used to increase uptake of the polio vaccine during campaigns and outreach sessions [13,21,24,25]. Overall, interventions to increase demand for childhood immunization and to improve uptake require holistic solutions that leverage health systems and community structures to promote the utility of immunization services for community health promotion. Participants also discussed the perceived positive effects that incentives or other free services would have on immunization-seeking behaviors. Non-cash incentives are frequently utilized to increase uptake of polio vaccine in northern Nigeria [26]. Application of small non-cash incentives to enhance uptake of childhood immunizations in various low- and middle-income countries has demonstrated temporary successes in improving coverage for a limited period. However, these incentives can be counterproductive when there is a risk of inconsistency in incentive supply chains [27,28]. In addition, consideration of incentives should be carefully considered alongside the finding that communities can be suspicious of the motivation behind free services.

### Strengths and limitations

One major strength of this study is the diverse range of participants from each LGA, including administrative staff, healthcare workers, community influencers, and caregivers. Inviting individuals with different community roles to participate in this rapid assessment helped to yield multifaceted, rich community-based perceptions of routine immunization in Sokoto state, Nigeria. However, there is always the possibility that important community perspectives were not included if some groups and individuals did not participate. This is a limitation of the current study, and of rapid assessments in general.ustained AFP surveillance in children younger than 15 years is critical.

## Conclusion

Despite intensified efforts to improve demand and uptake of health facility-based childhood immunizations in Sokoto state, findings from our assessment point to caregiver dissatisfaction with health facilities and immunization services, healthcare worker training gaps, gender dynamics, and rumors affecting perception and uptake of immunizations. As recommended by participants, social mobilization and community engagement strategies should be locally targeted to include the involvement of trusted leaders and community representatives. Even though incentives were viewed as a likely means to improve uptake, participants also recognized the potential for adverse consequences if such incentives are not sustained. Most of the barriers to receiving childhood immunizations found in our assessment reflect longstanding challenges facing the immunization program in northern Nigeria. Holistic interventions are needed to address underlying systems and socio-behavioral factors affecting low demand for and uptake of childhood immunization services in Sokoto state, Northwest Nigeria.

### What is known about this topic

- Gaps in immunization coverage continue to persist in Africa, with diphtheria-tetanus-pertussis (DTP3) uptake at 74% compared to 80% for other regions of the world;
- Nigeria has faced continued challenges achieving GVAP goals of ≥ 90% national coverage and ≥ 80% coverage in every district for all vaccines in a country´s national immunization schedule by 2020;
- Previous literature has explored factors for low vaccination demand, including vaccination rumors, lack of trust in vaccinations and fear of adverse events following immunizations. However, most of these studies were conducted in other Nigerian states, and did not look deeply at service delivery barriers.

### What this study adds

- This rapid qualitative assessment describes how social and behavioral drivers can explain health facility-based immunization coverage gaps among children in Sokoto state, Northwest Nigeria, which has been an unexplored area of study;
- The study offers recommendations from community members on how best to increase demand for and uptake of immunizations, and which strategies are likely to improve immunization service delivery to better address community needs;
- These findings can assist immunization program managers and other health managers and other health system staff, non-governmental organizations, multilateral institutions, and other key partners in Nigeria in improving immunization service delivery in Nigeria, thereby improving vaccine uptake. This study adds important information about social and behavioral drivers of under vaccination in Nigeria, which will advance the science and practice of increasing demand for immunization. system staff, non-governmental organizations, multilateral institutions, and other key partners in Nigeria in improving immunization service delivery in Nigeria, thereby improving vaccine uptake. This study adds important information about social and behavioral drivers of under vaccination in Nigeria, which will advance the science and practice of increasing demand for immunization.
